# Enhancing the diagnostic capacity of [^18^F]PSMA-1007 PET/MRI in primary prostate cancer staging with artificial intelligence and semi-quantitative DCE: an exploratory study

**DOI:** 10.1186/s41824-024-00225-5

**Published:** 2024-11-08

**Authors:** Philip Alexander Glemser, Martin Freitag, Balint Kovacs, Nils Netzer, Antonia Dimitrakopoulou-Strauss, Uwe Haberkorn, Klaus Maier-Hein, Constantin Schwab, Stefan Duensing, Bettina Beuthien-Baumann, Heinz-Peter Schlemmer, David Bonekamp, Frederik Giesel, Christos Sachpekidis

**Affiliations:** 1 Department of Radiology, German Cancer Research Center (DKFZ), Heidelberg, Germany; 2 Department of Nuclear Medicine, Faculty of Medicine, Medical Center-University of Freiburg, University of Freiburg, Freiburg, Germany; 3 Division of Medical Image Computing, German Cancer Research Center (DKFZ) Heidelberg, Heidelberg, Germany; 4 Medical Faculty, Heidelberg University, Heidelberg, Germany; 5 Clinical Cooperation Unit Nuclear Medicine, German Cancer Research Center (DKFZ), Im Neuenheimer Feld 280, D-69210 Heidelberg, Germany; 6 Department of Nuclear Medicine, University Hospital Heidelberg, Heidelberg, Germany; 7 Pattern Analysis and Learning Group, Department of Radiation Oncology, Heidelberg University Hospital, Heidelberg, Germany; 8 Institute of Pathology, Heidelberg University Hospital, Heidelberg, Germany; 9 Department of Urology, University Hospital Heidelberg, Heidelberg, Germany; 10 Section of Molecular Urooncology, Department of Urology, Medical Faculty Heidelberg, University Hospital Heidelberg, Heidelberg, Germany; 11 Department of Nuclear Medicine, Medical Faculty, University Hospital Duesseldorf, Düsseldorf, Germany

**Keywords:** ^18^F-PSMA-1007, PET/MRI, AI, DCE, Primary staging, Prostate cancer

## Abstract

**Background:**

To investigate the ability of artificial intelligence (AI)-based and semi-quantitative dynamic contrast enhanced (DCE) multiparametric MRI (mpMRI), performed within [^18^F]-PSMA-1007 PET/MRI, in differentiating benign from malignant prostate tissues in patients with primary prostate cancer (PC).

**Results:**

A total of seven patients underwent whole-body [^18^F]-PSMA-1007 PET/MRI examinations including a pelvic mpMRI protocol with T2w, diffusion weighted imaging (DWI) and DCE image series. Conventional analysis included visual reading of PET/MRI images and Prostate Imaging Reporting & Data System (PI-RADS) scoring of the prostate. On the prostate level, we performed manual segmentations for time-intensity curve parameter formation and semi-quantitative analysis based on DCE segmentation data of PC-suspicious lesions. Moreover, we applied a recently introduced deep learning (DL) pipeline previously trained on 1010 independent MRI examinations with systematic biopsy-enhanced histopathological targeted biopsy lesion ground truth in order to perform AI-based lesion detection, prostate segmentation and derivation of a deep learning PI-RADS score. DICE coefficients between manual and automatic DL-acquired segmentations were compared. On patient-based analysis, PET/MRI revealed PC-suspicious lesions in the prostate gland in 6/7 patients (Gleason Score-GS ≥ 7b) that were histologically confirmed. Four of these patients also showed lymph node metastases, while two of them had bone metastases. One patient with GS 6 showed no PC-suspicious lesions. Based on DCE segmentations, a distinction between PC-suspicious and normal appearing tissue was feasible with the parameters fitted maximum contrast ratio (FMCR) and wash-in-slope. DICE coefficients (manual vs. deep learning) were comparable with literature values at a mean of 0.44. Further, the DL pipeline could identify the intraprostatic PC-suspicious lesions in all six patients with clinically significant PC.

**Conclusion:**

Firstly, semi-quantitative DCE analysis based on manual segmentations of time-intensity curves was able to distinguish benign from malignant tissues. Moreover, DL analysis of the MRI data could detect clinically significant PC in all cases, demonstrating the feasibility of AI-supported approaches in increasing diagnostic certainty of PSMA-radioligand PET/MRI.

**Supplementary Information:**

The online version contains supplementary material available at 10.1186/s41824-024-00225-5.

## Introduction

Multiparametric MRI (mpMRI) including anatomic triplanar T2w-, diffusion-weighted- (DWI) and dynamic contrast-enhanced images (DCE) has established itself as the standard clinical imaging procedure for primary prostate cancer (PC) diagnosis and local staging (Mottet et al. 2021). In particular, the combination of mpMRI using PI-RADS (Prostate Imaging – Reporting and Data System) evaluation (Turkbey et al. 2019) and subsequent MR-transrectal ultrasound (MR-TRUS) guided targeted and systematic biopsies, implemented in many centers, has led to a higher PC detection rate, more accurate tumor grading, and improvement of T-staging (Siddiqui et al. 2013; Radtke et al. 2016; Bonekamp et al. 2019). With regard to DCE, although PI-RADS version 2.1 includes a binary assessment of early contrast enhancement of each lesion (Turkbey et al. 2019), an elaborate semi-quantitative DCE analysis is not part of the scoring system, which may underestimate the potential of DCE imaging (Bonekamp and Macura 2008; Tavakoli et al. 2023).

In the last decade hybrid PET/CT imaging with PSMA radioligands has become widely accepted as a robust imaging modality in the setting of PC biochemical relapse after radical prostatectomy or radiotherapy (Calais et al. 2019; Fendler et al. 2019; Giesel et al. 2019; Strauss et al. 2021). In addition, PSMA PET/CT is now considered a promising modality in the primary staging of PC (Hoffmann et al. 2022; Hofman et al. 2020; Emmett et al. 2021; Sachpekidis et al. 2016; Giesel et al. 2018; Sprute et al. 2021), although preoperative PSMA imaging is not yet standard practice in most institutions.

PSMA PET/MRI is a hybrid imaging modality that may aid in the diagnosis and staging of PC, combining the high performance of PSMA PET for whole body assessment with the multiparametric potential and high soft tissue contrast of MRI (Moradi et al. 2022; Evangelista et al. 2021). Despite initial promising results from its application, PET-MRI is not as widely used as PET/CT in the clinical routine of PC, mainly due to limited availability, increased cost, reduced patient throughput compared to PET/CT and long acquisition protocols (Beuthien-Baumann et al. 2021).

In our institution hybrid imaging with PSMA PET/MRI has been employed for several years for both primary staging and biochemical recurrence of PC (Freitag et al. 2016, 2018; Glemser et al. 2022). Moreover, novel deep learning pipelines have recently been developed for the standardized MRI approach (Schelb et al. 2019, 2021a, b; Bonekamp et al. 2018) although the value of artificial intelligence (AI) in PC diagnostics remains to be clarified. In this context, aim of the present exploratory study is to evaluate the ability of artificial intelligence (AI)-based and semi-quantitative dynamic contrast enhanced (DCE) multiparametric MRI (mpMRI), performed within [^18^F]-PSMA-1007 PET/MRI, in differentiating benign from malignant prostate tissues in patients with primary PC.

## Materials and methods

### Patients

A total of seven patients with a histopathological diagnosis of PC underwent whole-body [^18^F]-PSMA-1007 PET/MRI, including an mpMRI protocol, at our institution. Patient characteristics are shown in Table 1. This retrospective study was approved by the ethics committee of the University of Heidelberg (S-572/2021) and conducted in accordance to the declaration of Helsinki in its current form. No written informed consent was obtained due to the retrospective design of the study.

**Table 1:** Characteristics of the studied patients

Patient number	Age	PSA at the time of PET/MRI	Gleason score	miTNM classification (according to Eiber M 2018)	Visual PI-RADS	DL-PI-RADS	DICE coefficient (T2w + ADC union)	Applied [^18^F]-PSMA-1007 activity (MBq)
1	77	18.9	9	T4, N1b, M1b	5	0.993 = 5	0.441	166
2	55	63.8	7b	T3b, N1b, M0	5	0.988 = 5	0.433	239
3	64	5.2	9	T2u, N0, M0	5	0.999 = 5	0.696	194
4	72	5.4	9	T2u, N0, M0	5	0.949 = 5	0.561	176
5	48	4.0	6	T0, N0, M0	2	0.05 = 2	-	241
6	69	33.2	9	T3b, N1a, M0	5	0.42 = 4	0.139	229
7	70	63.0	9	T3b, N1b, M1b	5	0.45 = 4	0.382	242

*miTNM*, molecular imaging TNM; *PI-RADS*, Prostate Imaging Reporting and Data System; *DL*, deep learning; *T2w*, T2-weighted image; *ADC*, apparent diffusion coefficient; DICE, coefficient according to (Dice 1945)

### PET-MRI acquisition

PET-MRI examinations were performed using a dedicated PET-MRI system (3T Biograph mMR, Siemens Healthineers, Erlangen, Germany). The examinations started with a multiparametric pelvic MRI protocol including high-resolution three-dimensional T2w, DWI.

with several b-values (b0, b50, b1000, b1500 s/mm^2^) and contrast-enhanced dynamic acquisitions.

Approximately 2 h after i.v. injection of [^18^F]-PSMA-1007, whole-body PET and MRI data were acquired simultaneously. Specifically, the PET data (3 min per bed position) were reconstructed using an iterative 3-D OSEM algorithm with two iterations, 21 subsets, 3 mm Gaussian filter and an image matrix of 344, µ-map FOV. The MRI acquisition protocol included whole body morphological axial and coronal T2w HASTE sequences.

### Visual (qualitative) analysis

PET/MRI data were visually analysed in consensus by a board-certified radiologist (PG, 9 years of experience in prostate imaging) and nuclear medicine physician (CS, 9 years of experience in PSMA PET imaging). Primary prostate tumors were analysed multiparametrically according to the current PIRADS classification on all available MRI sequences and on the basis of increased radiotracer uptake relative to normal background prostate parenchyma. Lymph nodes were considered suspicious on the basis of increased tracer uptake and/or MRI features such as short-axis diameter > 8 mm (pelvis) or > 10 mm (other regions), marked diffusion restriction, spherical configuration, inhomogeneity, etc. according to (McMahon et al. 2010; Sawicki et al. 2019). Distant metastases were defined as lesions with increased [^18^F]-PSMA-1007 uptake (with disregard of known benign PSMA-avid structures e.g., ganglia, ureters), taking into account the respective morphological findings in MRI (Sheikhbahaei et al. 2019).

### Quantitative analysis

#### DCE analysis

Segmentations were performed manually with the software framework Medical Imaging Interaction Toolkit (MITK, version 2018.4.0; https://docs.mitk.org/2018.04/index by a PhD trainee (BK, 3 years experience in medical imaging analysis) and a board certified radiologist (PG) under the supervision of a board-certified radiologist and prostate MRI specialist (DB, 13 years of experience in prostate imaging). Tumor suspicious lesions (TSL) were segmented on the axial DCE phases with optimal contrast enhancement typically observed in the phases of wash-in timepoint and 1–2 timepoints thereafter; for curve discrimination we segmented normal appearing tissue (NAT) in DCE in peripheral zone of the prostate according to (Tavakoli et al. 2023). To support our segmentation validity, segmentations of perilesional tissue (PLT) directly adjacent to the tumor suspicious lesions were added as well to be analysed in DCE. Segmentations were used to calculate the intensity values for each time-point by averaging the voxel-intensity values of each tissue. Based on those calculated time-intensity curves, complex DCE ratios between the segmented tissues (TSL/NAT and TSL/PLT) were calculated using common DCE characteristic features (Tavakoli et al. 2023; Petralia et al. 2020).

Quantitative DCE parameters are depicted in Table 2 and detailed in Suppl. Table 1.

**Table 2:** DCE characteristics for patients with suspicious primary prostate lesions, excluding patient 5 with no suspicious primary lesion. Detailed information on all DCE parameters investigated can be found in supplementary table 1

Patients		1	2	3	4	6	7
Wash-in ratio	TSL/NAT	3.27	4.17	1.57	5.91	4.21	2.12
	TSL/PLT	1.94	3.40	1.70	1.41	2.64	1.85
Wash-out ratio	TSL/NAT	0.63	-1.21	-0.12	1.55	-0.07	1.24
	TSL/PLT	0.58	-0.79	-0.12	0.83	-0.04	0.74
Fitted maximum value	NAT	1.00	1.00	0.98	1.01	0.98	0.95
	PLT	2.16	1.16	1.07	5.36	1.41	1.44
	TSL	4.22	3.01	1.46	10.61	3.26	3.27
fMCR	TSL/NAT	4.22	3.01	1.49	10.45	3.32	3.46
	TSL/PLT	1.95	2.60	1.36	1.98	2.30	2.27

*TSL*, tumor suspicious lesion; *NAT*, normal appearing tissue (segmentation in peripheral zone of the prostate); *PLT*, perilesional tissue (segmentation directly adjacent to the tumor suspicious lesion); *fMCR*, fitted maximum contrast ratio

#### AI-supported analysis of MRI data

The co-registration and deep learning pipeline used in our study cohort is identical to (Netzer et al. 2021) and was previously trained on 1010 single-vendor multi-scanner (one 1.5 Tesla and four 3.0 Tesla MRI scanners) examinations. It used T2w, high b-value images and ADC maps as input. In this study, the model was used for inference only, without any additional training taking place.

Briefly, each lesion in the training cohort was assigned a systematic biopsy enhanced lesion histopathological ground truth (SELGT) determined by the highest Gleason score (GS) from both targeted and overlapping sextant systematic biopsy cores. Lesion segmentations with a GS ≥ 7a were selected for model training.

The raw T2w and DWI/ADC DICOM images were converted to NIFTI format and up-sampled to match the resolution of the T2w image via third-order spline interpolation. A multi-resolution, multi-step process was used to register diffusion-weighted images (b0 or b50 DWI) to T2-weighted images, involving a sequence of rigid, affine, and elastic transformations as described in (Netzer et al. 2021). The resulting transformation was applied to high b-value images and ADC maps.

After registration, the images were cropped to concentrate the CNN training on the prostate and to allow for variable FOVs. A size-dependent cropping approach was employed to ensure that prostate size was preserved as a feature for the CNN. The median dimensions of the input images for nnUNet were 297 × 261 × 21 in the x/y/z axes.

To create an ensemble of ten models, initial training involved an 80%/20% random split of the available datasets in five-fold cross-validation, which was used to train five 2D and five 3D U-Nets. nnUNet analyzed the dataset for optimal image preprocessing and network architectures. Each input image was individually z-scored and the ADC maps were globally normalized. The network architecture was tailored to cover the entire input patch.

The ten U-Nets each generated three-dimensional tumor probability maps corresponding to the dimensions of the input images. These maps were averaged to generate the final prediction, with each voxel assigned a value ranging from zero to one, indicating the likelihood of a suspicious finding. The first 300 examinations of the original test cohort were used to calibrate deep learning PIRADS scores (DL-PIRADS 3–5) corresponding to clinically observed radiologist operating points. They were defined by using cut-off values for model predictions to achieve 97%/90% sensitivity and 90% specificity in the 300 exams, respectively.

The calibrated DL-PIRADS predictions were 0.033 for PIRADS 2, 0.074 for PIRADS 3, 0.186 for PIRADS 4 and 0.646 for PIRADS 5.

## Results

### PET/MRI visual analysis

**Fig. 1:**
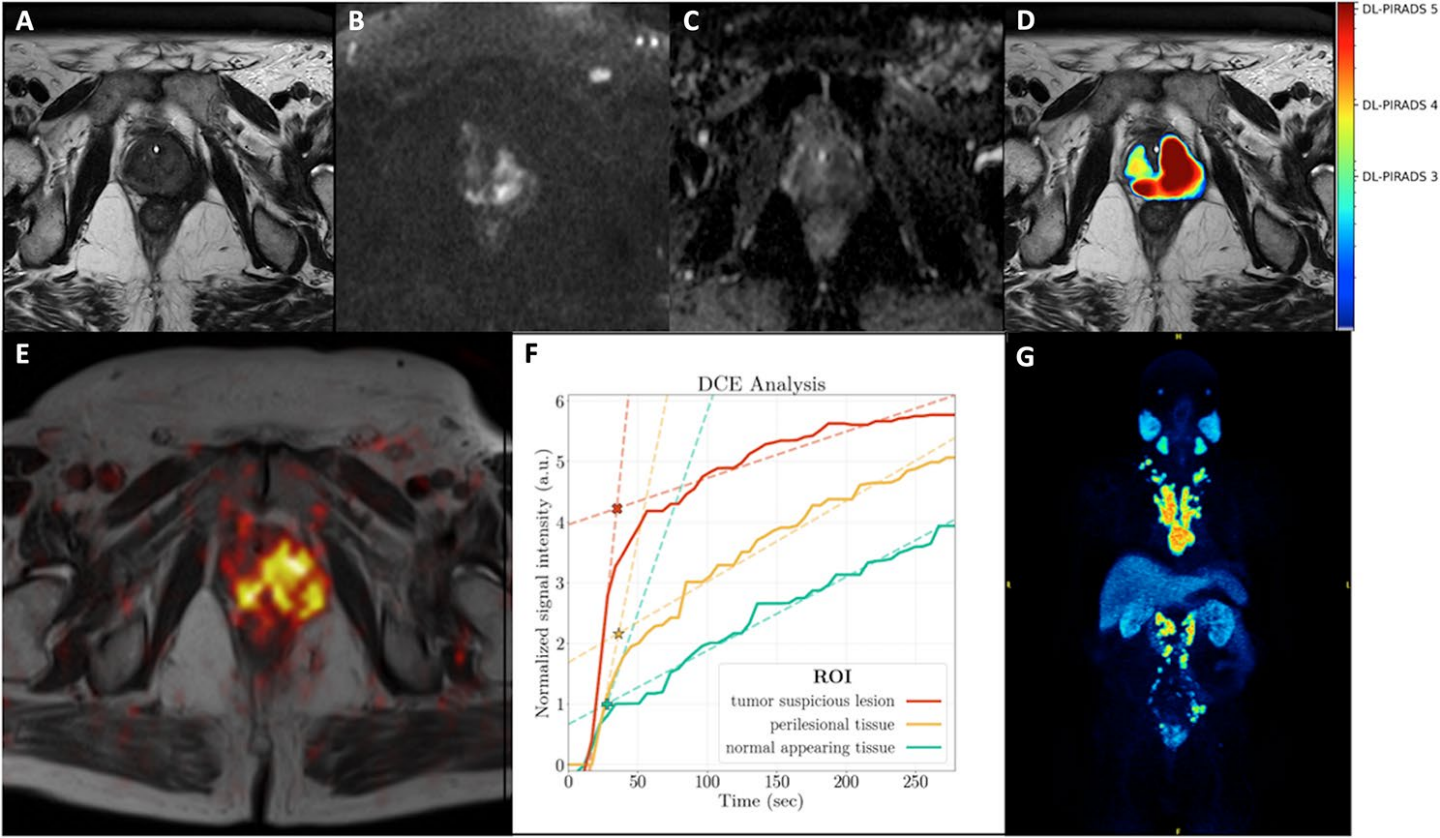
A 77-year-old patient with an initial diagnosis of PC (Gleason score 9) referred for initial staging with PET-MRI. His PSA level at the time of examination was 18.9 ng/ml. Distinct T2w hypointensity of the prostate (**A**) with marked diffusion restriction with signal increase in the high b-values (**B**) and low ADC values in the ADC-map (**C**) predominantly in the left hemisphere of the prostate with extension over the midline to the right side and with extracapsular extension compatible with a PIRADS score of 5. The AI-based PI-RADS equivalent (DL-PIRADS) is in good agreement with the MRI findings and also shows a very high probability score for PCa (DL-PIRADS 5, **D**). Correspondingly, [^18^F]-PSMA-1007 PET (fused T2w, (**E**)) shows an intense [^18^F]-PSMA-1007 accumulation in the prostate gland. Quantitative DCE data based on manual segmentation showed a steeper wash-in slope for tumor-suspicious lesion (TSL, red) compared to perilesional tissue (PLT, yellow) and normal appearing tissue (NAT, green). The corresponding fitted maximum (*, intersection of wash-in and wash-out slope) was 4.2 for TSL, 2.2 for PLT and 1.0 for NAT, allowing a clear discrimination between them. All 3 curves showed an increasing curve pattern (**F**). Whole-body [^18^F]-PSMA-1007 PET imaging showed the primary tumor, multiple iliac, retroperitoneal, mediastinal and cervical lymph node metastases, as well as bone metastases in the sacral bone and the thoracic spine (**G**)

**Fig. 2:**
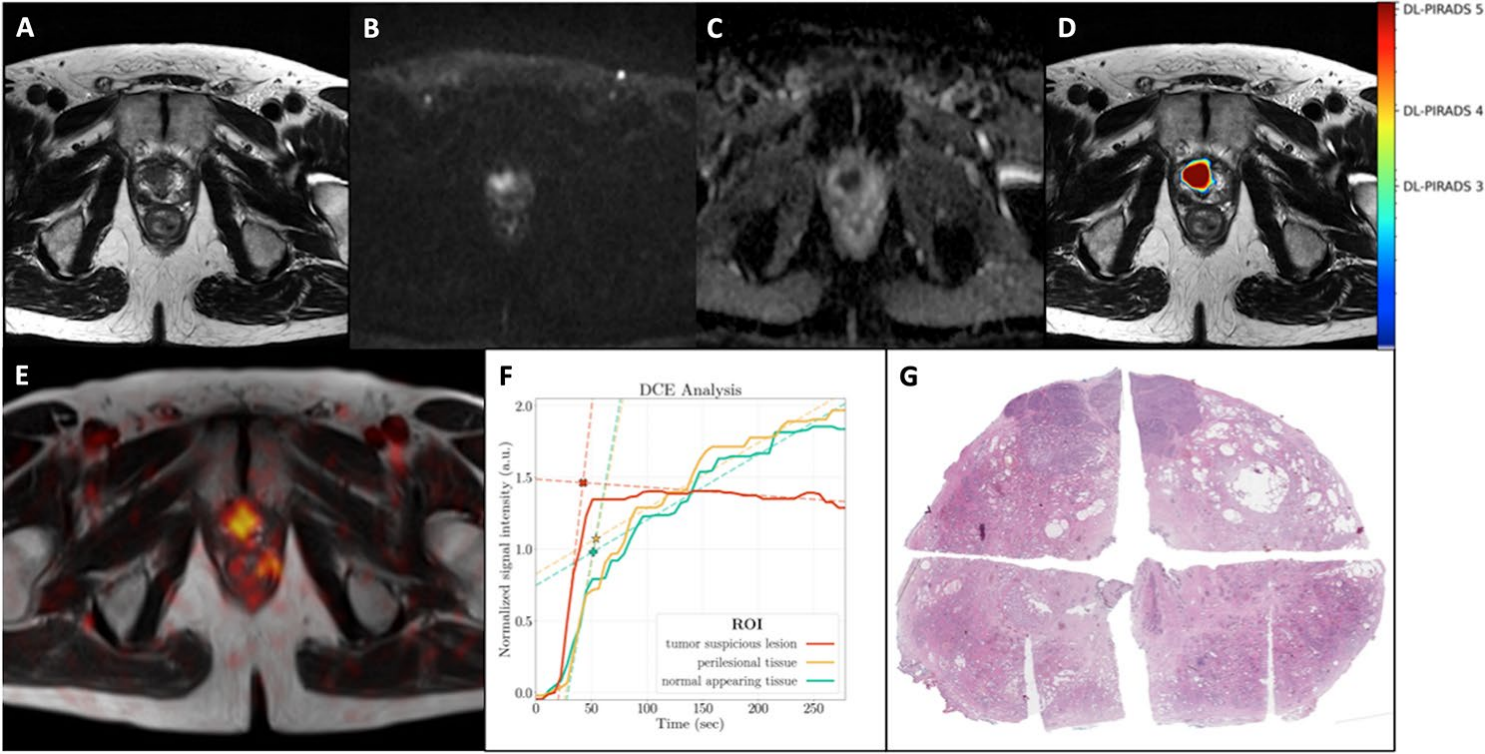
A 64-year-old patient with an initial diagnosis of PC (Gleason score 9) referred for initial staging with PET-MRI. His PSA level at the time of examination was 6.8 ng/ml. MRI showed a PC suspicious lesion in the anterior peripheral and adjacent transitional zone in the predominatly right-sided prostate apex with a diameter of 16 mm, showing T2w hypointensity (**A**), marked diffusion-restriction with signal enhancement in the high B-values (**B**) and signal decrease in the ADC-map (**C**), compatible with PIRADS 5 score. The AI probability score is in good agreement with the MRI findings, which also show a very high probability score for PC (DL-PIRADS 5, **D**). PET-MRI (fused T2w, **E**) shows intense [^18^F]-PSMA-1007 accumulation in this lesion. Quantitative DCE data based on manual segmentation showed a steeper wash-in-slope for the tumor-suspicious lesion (TSL, red) compared to the perilesional tissue (PLT, yellow) and normal appearing tissue (NAT, green), which showed comparable wash-in slopes. The washout slope for TSL was slightly negative. PLT and NAT showed an increasing curve pattern. The corresponding fitted maximum (*, intersection of wash-in and wash-out slopes) was 1.5 for TSL, 1.1 for PLT and 1.0 for NAT, allowing a clear distinction between TSL versus PLT and NAT (**F**). Whole-body [^18^F]-PSMA-1007 PET imaging (not shown) showed no lesions consistent with metastases. Histology confirmed an anterior, predominantly right-sided prostate carcinoma (digitally merged whole-mount prostate slide, hematoxylin and eosin staining, **G**)

**Fig. 3:**
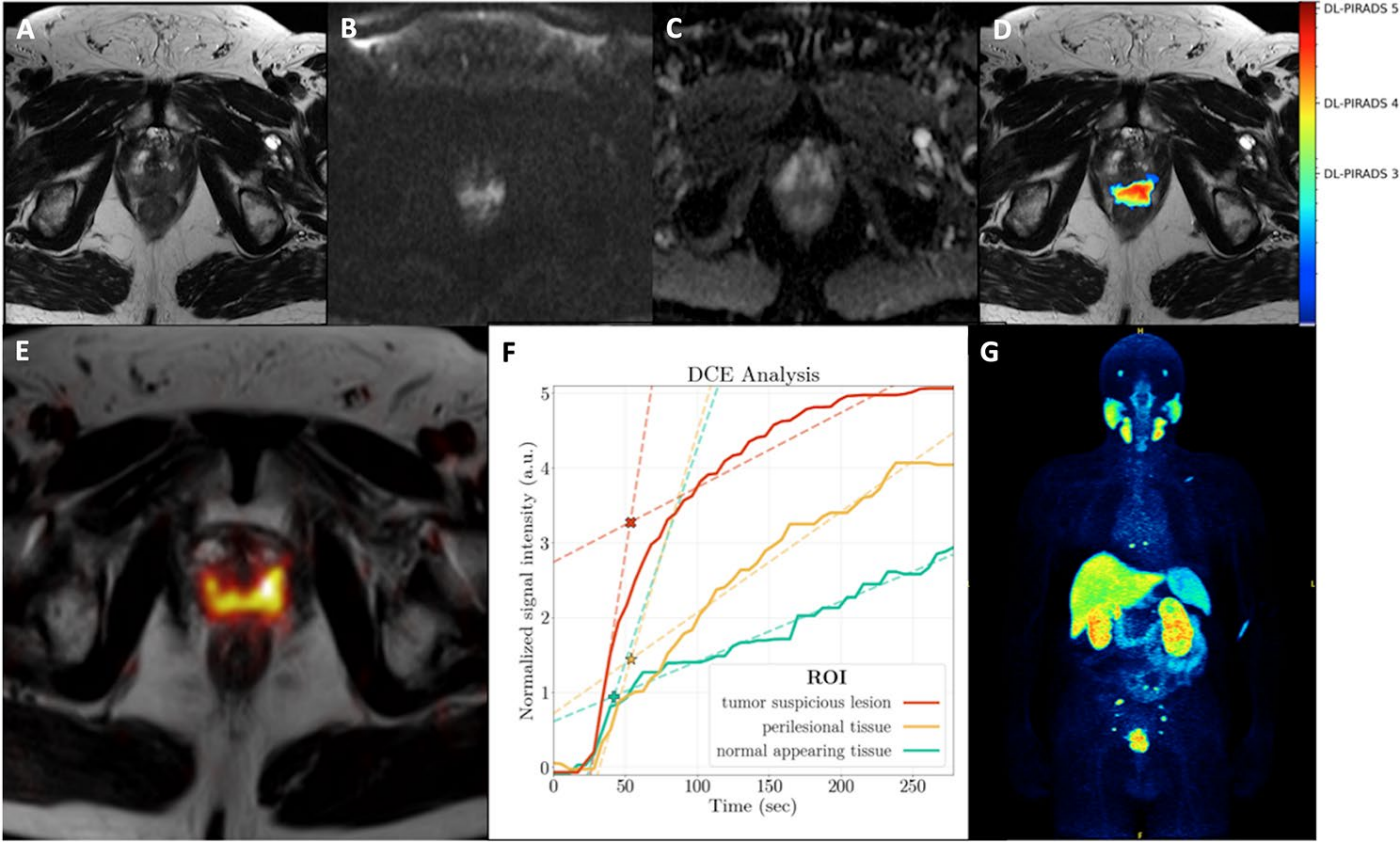
A 70-year-old patient with an initial diagnosis of PC (Gleason score 9) referred for initial staging with PET-MRI. His PSA level at the time of examination was 63.0 ng/ml. MRI showed a lesion suspicious for PC mainly in the posteromedial/-lateral peripheral zone with infiltration in the seminal vesicles (not shown) with T2w hypointensity (**A**) and diffusion restriction with increase in high b-value (**B**) and decrease in ADC map (**C**). The AI probability score is in good agreement with the MRI findings and also shows a high probability score for PC (DL-PIRADS 4, **D**). Quantitative data of DCE based on manual segmentations showed a steeper wash-in slope for tumor-suspicious lesion (TSL, red) compared to perilesional tissue (PLT, yellow) and normal appearing tissue (NAT, green). The corresponding fitted maximum (*, intersection of wash-in and wash-out slope) was 3.3 for TSL, 1.4 for PLT and 0.9 for NAT, enabling a clear distinction between TSL, PLT and NAT. All three curves showed an increasing curve pattern (**F**). Whole-body [^18^F]-PSMA-1007 PET imaging revealed the primary tumor as well as multiple PSMA-avid iliac lymph node metastases and osseous metastases in the sacral bone, thoracic spine and left scapula (**G**)

In six out of seven patients, PET/MRI demonstrated at least one PC suspicious lesion in the prostate gland. These patients had a GS of ≥ 7b. Moreover, two of these patients showed lymph nodes suspicious for metastatic involvement, while two patients had findings suspicious for both lymph node and bone metastases. One patient (patient 5, GS 6) had no lesion suspicious for PC manifestations (either primary or metastatic). Some patient examples are shown in Figs. 1, 2 and 3.

### DCE quantitative results of prostate lesions

For all patients included, our data show consistently higher fitted maximum values for TSL compared with PLT and NAT. Wash-out slopes based on detected TSL showed negative values in three of six cases. Moreover, the combination of wash-in- and wash-out slopes for the fitted maximum values could discriminate tumor versus NAT (Suppl. Table 1). Further, with concern to quantitative DCE ratio parameters, wash-in ratio and fMCR were discriminative in all cases. The results of the quantitative DCE analysis can be found in Table 2.

### AI analysis

In 5/7 patients, visual and DL-PIRADS showed consistent scores (Pat. 1–5). In two patients (patients 6 and 7) visual PIRADS gave a score of 5 while DL-PIRADS gave a score of 4. The corresponding DICE scores DL- vs. manual segmentation were > 0.38 in five patients and lower in one patient (patient 6) (Table 1). All patients with a DL-PI-RADS score ≥ 4 had a Gleason score of ≥ 7a (clinically significant PC).

## Discussion

In this exploratory study we investigated the ability semi-quantitative DCE- and AI-based analysis procedures integrated within hybrid [^18^F]-PSMA-1007 PET/MRI in distinguishing benign from malignant tissues in primary prostate PC. Besides confirming the ability of [^18^F]-PSMA-1007 PET/MRI to detect local and distant PC lesions, semi-quantitative DCE analysis based on manual segmentation of time intensity curves for tumor suspicious, peritumoral and normal appearing tissue was able to discriminate benign from malignant tissue. Furthermore, AI-based analysis of the MRI data (bimodal AI approach with T2w and DWI/ADC) correctly detected clinically significant PC in all patients according to biopsy results, with moreover a good correlation between manual and automatic AI segmentations.

[^18^F]-PSMA-1007 PET/MRI was able to detect PC lesions (primary tumors and metastases) in 6/7 patients of the studied cohort, while one patient did not show any suspicious lesions, which may be however attributed to his GS of 6. This result is in line with the prospective study by Ferraro et al., who performed PET/MRI-guided biopsies in 42 PC patients, and reported a patient-based sensitivity, specificity and accuracy for significant PC of 96%, 81%, and 90%, respectively, with only one patient being diagnosed with a PSMA-negative PC (Ferraro et al. 2021). In another prospective study of men with a new diagnosis of intermediate- or high-grade PC who underwent [^68^Ga]-PSMA-11 PET/MRI, 72/73 enrolled patients had focal uptake in the prostate, while PSMA-avid metastatic disease was identified in 20 of them. Moreover, tracer uptake correlated with grade group and PSA, while a high uptake in the primary tumor and the presence of PSMA metastases were associated with biochemical failure, highlighting the ability of the in risk-stratifying patients with intermediate- or high-grade PC prior to prostatectomy (Hoffmann et al. 2022). Our results underline the potential role of [^18^F]-PSMA-1007 PET/MRI as a powerful staging tool, especially in the setting of initial staging of intermediate- or high-grade PC.

DCE has been a part of the standard mpMRI acquisition, helping to differentiate between malignant and benign tissues based on its ability to assess tumor angiogenesis (Engelbrecht et al. 2003) through early and pronounced tumor contrast enhancement. This was taken into account in its use in the outdated first version of PI-RADS V1 (Petralia et al. 2020), in which manually ROI-based lesion DCE signal intensity time curves (type 1: slow initial curve increase, persistent enhancement; type 2: medium initial curve increase with reaching of a plateau; type 3: fast initial curve increase, rapid washout) were included equivalent to DWI and T2w sequences, resulting in a summed score indicating the respective PIRADS score. However, the enhancement patterns of PC are heterogeneous and it hasn’t been possible to maintain the use of these three specific curve types in daily routine. Therefore, the role of DCE has been downgraded in the new version of PIRADS (Turkbey et al. 2019). However, PI-RADS version 2.1 remains multiparametric with the inclusion of DCE, perhaps also taking into account the particular value of DCE when T2w or DWI sequences are missing due to motion or extinction artefacts in the pelvis, or in tricky cases where all available image information is indispensable for a comprehensive case solution.

**Fig. 4:**
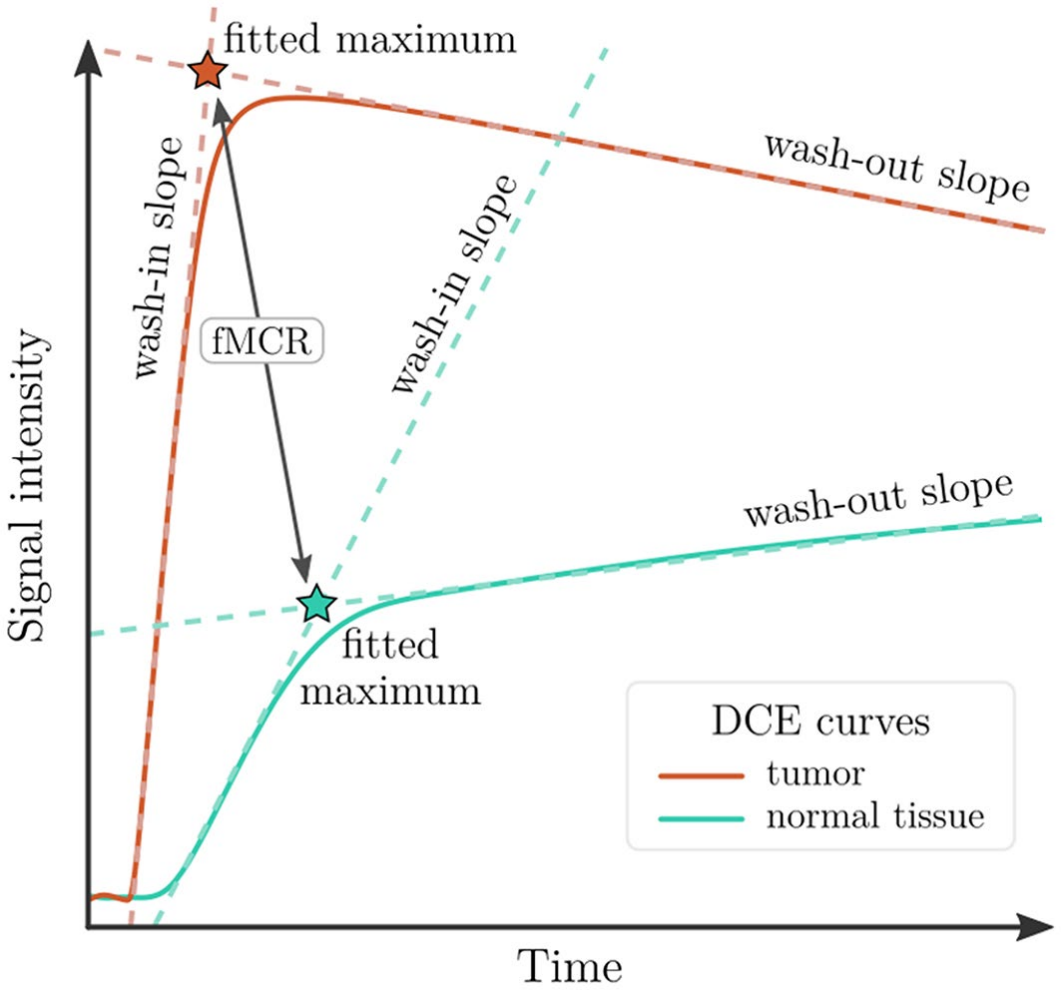
Schematic representation of DCE features for quantitative differentiation of malignant and normal prostate tissue conditions. The wash-in and wash-out slopes of an intensity curve (tumor or normal tissue) can be used to determine each “fitted maximum” (intersection of the dashed lines, shown here with red and green asterisks). The ratio of these fitted maximum intensity values is called “fitted maximum contrast ratio” (fMCR)

In recent years, semi-quantitative models and parameters have been discussed by several authors and the respective time-intensity curves have been de-constructed into curve parts or features and presented as “wash-in slope”, “wash-out slope” or “fitted maximum” (Fig. 4), allowing relative comparisons, i.e. between tumor-suspicious and normal tissue (Tavakoli et al. 2023; Petralia et al. 2020; Medved et al. 2004; Tuncbilek et al. 2005). In our cohort, several DCE parameters were applied in the same way. The best predictive parameter was the fitted maximum contrast value and/or the fitted maximum contrast ratio between tumor and normal-appearing (as well as tumor vs. peritumoral and peritumoral vs. normal tissue), which was discriminatory for the whole cohort, while the wash-in ratio performed equally well. fMCR was able to discriminate tumor from non-tumor even in a “prebiopsied” study case (Fig. 2), where other DCE parameters such as maximum value or AUC failed due to contrast drain-off at the lesion site over time. In conclusion, the above DCE parameters fit well with the complementary methods presented in this article for the detection of significant PC.

More recently, the field of AI in prostate imaging has emerged with the long-term goal of objectifying prostate assessment, facilitating and accelerating workflow, and improving diagnostic performance for the patient. Currently, almost all of these pipelines are in advanced development or in simulated clinical use (Schelb et al. 2021a, b). Previous AI studies remain limited due to the small size of training or test cohorts, the lack of adequate systematic histopathological ground-truth mapping of the prostate (i.e. by using targeted but non-systematic biopsies or even relying on PIRADS alone without biopsy-proven ground-truth), or the unavailability of a fully automated DL approach (Arif et al. 2020; Winkel et al. 2021; Deniffel et al. 2020). In an attempt to address these issues, Netzer et al. investigated the diagnostic performance of different U-Nets with extended biopsy ground truth (Radtke et al. 2016; Netzer et al. 2021) as a function of the size of the training cohort and the addition of bi-institutional data to some cohorts. Thereby, a large training cohort with 1010 data sets showed very promising results by receiver operating characteristics with an AUC of 0.85. This model outperformed another smaller cohort in the same study with 171 training data sets in terms of patient-based and sextant-based predictive performance. The larger training set was applied to our patient cohort (Table 1) and performed well in detecting every significant PC in our study, although the training pipeline saw the fewest datasets in the training cohort on the Biograph MRI scanner (33 out of 1010). One patient (Patient 5) was graded as a DL-PI-RADS of 2, the subsequent biopsy showed a non-significant PC with a Gleason score of 6 thus DL approach seemed to discriminate significant PC and non-significant PC in our cohort. Two patients in our study (Patient 6 and 7) were slightly underestimated by the DL pipeline, giving a DL-PIRADS of 4, whereas visual analysis had detected PIRADS 5, but this wouldn’t have led to a change in clinical management. The DICE coefficients between manual and deep learning segmentation had a mean value of 0.44, comparable to the original cohort (Netzer et al. 2021) (with 0.42/0.53 for PI-RADS 3/4 equivalents) and even to manual segmentation correlations between different radiologists with a mean value of 0.48–0.52 (Schelb et al. 2021a, b). In summary, the DL pipeline used can serve as an additional “virtual radiologist” reader.

Our study has some limitations. The cohort studied was small with seven patients, so further studies with larger numbers of patients are needed in the future to confirm the performance of DCE and AI concerning the primary tumor. However, regarding the application of AI, the initial training cohort was one of the largest ever published with > 1000 datasets. Concerning whole body staging, another limitation is that lymph node and distant metastases were not histopathologically validated. However, this is not the case in the clinical setting. In addition, previous studies have shown a high correlation between imaging and histopathological findings for PSMA tracers (Hofman et al. 2020; Maurer et al. 2016; Rahman et al. 2019).

## Conclusion

We investigated the role of [^18^F]-PSMA-1007 PET/MRI taking into account additional semi-quantitative DCE- and AI-based analysis procedures in primary PC diagnostics. In terms of T-staging, an MRI-based deep learning pipeline based on a large training data cohort as well as semi-quantitative DCE parameters such as fMCR were able to delineate significant PC lesions and discriminate them from normal prostate tissue. A combination of the methods presented here may help to improve the diagnostic accuracy of PC.

## Electronic supplementary material

Below is the link to the electronic supplementary material.

Supplementary Material 1

## Data Availability

The datasets generated during and/or analysed during the current study are available from the corresponding author on reasonable request.
