# Supplementary material for: Enhancing the diagnostic capacity of [18F]PSMA-1007 PET/MRI in primary prostate cancer staging with artificial intelligence and semi-quantitative DCE: an exploratory study
Source: EJNMMI Rep. 2024 Nov 8;8(1):37. doi: 10.1186/s41824-024-00225-5 (PMC11543981; doi:10.1186/s41824-024-00225-5)
Supplement: Supplementary file 1 — Supplementary Material 1 [file 41824_2024_225_MOESM1_ESM.docx]

**Supplementary Table 1** DCE characteristics for patients with suspicious primary lesions of prostate, Patient 5 with no suspicious primary lesion excluded.

| **Patients** |  | **1** | **2** | **3** | **4** | **6** | **7** |
| --- | --- | --- | --- | --- | --- | --- | --- |
| **Wash-in slope** | NAT | 0.067 | 0.048 | 0.043 | 0.095 | 0.036 | 0.057 |
|  | PLT | 0.112 | 0.058 | 0.040 | 0.401 | 0.058 | 0.066 |
|  | TSL | 0.218 | 0.199 | 0.068 | 0.563 | 0.152 | 0.122 |
| **Maximum value** | NAT | 3.933 | 1.380 | 1.853 | 4.688 | 1.883 | 2.932 |
|  | PLT | 5.059 | 1.824 | 1.966 | 12.208 | 3.106 | 4.065 |
|  | TSL | 5.763 | 2.837 | 1.403 | 15.282 | 3.237 | 5.058 |
| **Wash-out slope** | NAT | 0.012 | 0.002 | 0.005 | 0.014 | 0.005 | 0.008 |
|  | PLT | 0.013 | 0.003 | 0.005 | 0.027 | 0.008 | 0.013 |
|  | TSL | 0.008 | -0.002 | -0.001 | 0.022 | 0.008 | 0.010 |
| **Fitted maximum value** | NAT | 1.000 | 1.000 | 0.980 | 1.015 | 0.983 | 0.947 |
|  | PLT | 2.165 | 1.160 | 1.072 | 5.362 | 1.415 | 1.445 |
|  | TSL | 4.222 | 3.012 | 1.462 | 10.608 | 3.260 | 3.272 |
| **Wash-in ratio** | TSL/NAT | 3.266 | 4.167 | 1.569 | 5.909 | 4.212 | 2.125 |
|  | TSL/PLT | 1.941 | 3.398 | 1.702 | 1.406 | 2.638 | 1.847 |
| **Wash-out ratio** | TSL/NAT | 0.631 | -1.207 | -0.123 | 1.552 | -0.075 | 1.244 |
|  | TSL/PLT | 0.576 | -0.790 | -0.123 | 0.834 | -0.042 | 0.741 |
| **AUC ratio** | TSL/NAT | 2.017 | 2.418 | 0.965 | 4.766 | 2.258 | 2.238 |
|  | TSL/PLT | 1.382 | 1.964 | 0.920 | 1.542 | 1.347 | 1.524 |
| **fMCR** | TSL/NAT | 4.222 | 3.012 | 1.491 | 10.451 | 3.317 | 3.455 |
|  | TSL/PLT | 1.950 | 2.596 | 1.364 | 1.978 | 2.305 | 2.265 |

TSL, tumor suspicious lesion; PLT, perilesional tissue (segmentation directly adjacent to the tumor suspicious lesion); NAT, normal appearing tissue (segmentation in peripheral zone of the prostate); AUC, area under the curve; fMCR, fitted maximum contrast ratio.
